# National Data on Age Gradients in Well-being Among US Adults

**DOI:** 10.1001/jamapsychiatry.2022.2473

**Published:** 2022-08-24

**Authors:** Ying Chen, Richard G. Cowden, Jeffery Fulks, John F. Plake, Tyler J. VanderWeele

**Affiliations:** 1 Department of Epidemiology, Harvard T.H. Chan School of Public Health, Boston, Massachusetts; 2 Human Flourishing Program, Institute for Quantitative Social Science, Harvard University, Cambridge, Massachusetts; 3 American Bible Society, Philadelphia, Pennsylvania; 4 Evangel University, Springfield, Missouri

## Abstract

This survey study assesses well-being among US adults by age group.

There have been increasing concerns about the well-being of young people in the US,^1,2^ but evidence has focused on mental health.^1^ Taking a comprehensive approach to well-being, we used data from a nationally representative sample of US adults to examine well-being scores by age group across numerous domains.

## Methods

The sample for this survey study was drawn from the NORC AmeriSpeak panel, a probability-based panel designed to be representative of the US household population.^3^ A stratified sample was selected based on age, race and ethnicity, gender, and education. Surveys were conducted online and via telephone using a 15-minute questionnaire. Data were collected from January 10 to 28, 2022. Based on US census data, post hoc weighting was performed to ensure the sample was representative of US adults 18 years or older within all 50 states and the District of Columbia. Participants responded to a validated 12-item measure of flourishing^4,5^ that assesses 6 domains of well-being, including happiness, health, meaning, character, relationships, and financial stability. Items were self-reported on a scale of 0 to 10. Domain scores were derived by calculating the mean of the 2 indicators under each domain; the overall well-being score was the mean indicator responses across domains. Ethical approval was granted by the NORC institutional review board. Participants provided written informed consent. This study followed AAPOR and STROBE reporting guidelines. Analysis of variance and the *t* test (2-sided) were used to examine group differences by age, race and ethnicity, and gender. Analyses were conducted using SAS, version 9.4. The eMethods in the Supplement gives more details.

## Results

Of 8618 individuals contacted, 2598 (30.1%) provided complete responses (1338 [51.50%] female; mean [SD] age, 47.92 [17.94] years). Well-being increased monotonically cross-sectionally with age for overall well-being, the domains, and across all individual items (Table and Figure). Differences between the youngest (age 18-25 years) and oldest (age ≥77 years) age groups were present for meaning (2.08 points; 95% CI, 1.63-2.53), happiness (1.99 points; 95% CI, 1.60-2.38), health (1.93 points; 95% CI, 1.56-2.31), relationships (1.91 points; 95% CI, 1.46-2.36), financial stability (1.83 points; 95% CI, 1.23-2.43), and character (1.28 points; 95% CI, 0.93-1.62). The maximum difference in overall well-being among age groups was between the youngest and oldest (1.84 points; 95% CI, 1.52-2.15) and was larger than the largest differences among Asian (mean [SD], 6.50 [2.27]), Black (mean [SD], 6.76 [1.78]), Hispanic (mean [SD], 6.67 [1.54]), White (mean [SD], 7.00 [1.50]), and other (≥2 races, non-Hispanic; other, non-Hispanic) (mean [SD], 6.53 [1.21]) race and ethnicity and between men (mean [SD], 6.87 [1.53]) and women (mean [SD], 6.88 [1.59]).

**Table.** Weighted Overall Well-being Score by Age Group in a Nationally Representative Sample of US Adults in January 2022^a^

Age group	Sample size	Well-being score	
		Mean (SD)	Median (IQR)
Gen Z (18-25 y)	331	6.14 (2.03)	6.17 (2.22)
Millennial (26-41 y)	734	6.59 (1.44)	6.67 (2.25)
Gen X (42-57 y)	656	6.83 (1.70)	7.08 (2.17)
Boomer (58-76 y)	757	7.32 (1.29)	7.42 (1.67)
Silent generation (≥77 y)	120	7.98 (1.01)	8.08 (1.50)
All	2598	6.87 (1.56)	7.08 (2.08)

^a^ The overall well-being score (measured with the secure flourishing index^4^; range, 0-10) is a mean score across 6 domains, including happiness and life satisfaction, mental and physical health, meaning and purpose, character and virtue, close social relationships, and financial and material stability. Age groups were created based on birth cohorts. Post hoc weighting was performed based on US census data to ensure the sample was representative of US adults 18 years or older within all 50 states and the District of Columbia. Multiple imputation was used to impute missing data on the well-being scores, and there were no missing data on age groups.

**Figure.**
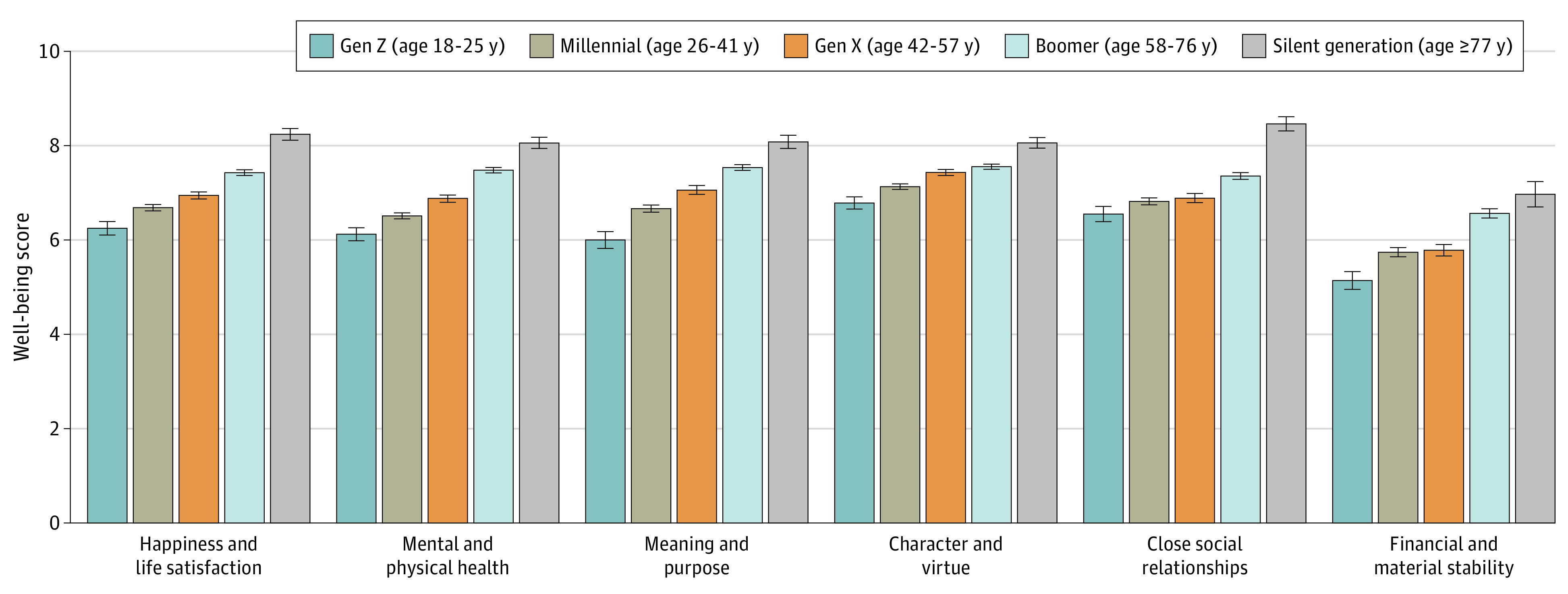
Weighted Domain-Specific Well-being Scores by Age Group in a Nationally Representative Sample of US Adults in January 2022 Age groups were created based on birth cohorts. Post hoc weighting was performed based on US census data to ensure the sample was representative of US adults 18 years or older within all 50 states and the District of Columbia. Multiple imputation was used to impute missing data on well-being scores, and there were no missing data on age groups. Error bars represent SEs.

## Discussion

This study found that mean well-being scores across multiple domains increased cross-sectionally with age, with a substantial age gradient. This finding contrasts with evidence from the early 2000s that showed U-shaped curves for some well-being domains (eg, happiness, life satisfaction), with well-being scores being higher in earlier adulthood and older age than in midlife.^6^ Our findings support evidence of a mental health crisis and increase in loneliness in the US that has disproportionally affected young adults^1,2^ and extend evidence of age gradients to multiple additional facets of well-being beyond mental health. Younger adults reported lower well-being even on the self-rated physical health item.

A limitation is that we could not disentangle age and cohort effects. Further studies are needed to replicate the findings. Although indicators in the study were obtained by self-report, this was also the case with earlier research that documented U-shaped patterns with age.^6^ These findings suggest that the well-being of young people has declined compared with older age groups. Protecting the mental health of young people is regarded as a national emergency^1^; this study suggests that other facets of their well-being also need attention. Comprehensive strategies for expanding mental health services, supporting education and meaningful employment, and strengthening social fabric for young people are needed to enhance the well-being of this population.

## Notes

Supplement.
eMethods
