# Supplementary material for: National Data on Age Gradients in Well-being Among US Adults
Source: JAMA Psychiatry. 2022 Aug 24;79(10):1046–7. doi: 10.1001/jamapsychiatry.2022.2473 (PMC9403847; doi:10.1001/jamapsychiatry.2022.2473)
Supplement: Supplement. — eMethods [file jamapsychiatry-e222473-s001.pdf]

Supplemental Online Content

Chen Y, Cowden RG, Fulks J, Plake JF, VanderWeele TJ. National data on age gradients in well-being among US adults. *JAMA Psychiatry*. Published online August 24, 2022. doi:10.1001/jamapsychiatry.2022.2473

eMethods

This supplemental material has been provided by the authors to give readers additional information about their work.

© 2022 Chen Y et al. *JAMA Psychiatry.*

eMethods

Study Sample

The sample for this study was drawn from the NORC at the University of Chicago AmeriSpeak panel, a probability-based panel designed to be representative of the U.S. household population. Details about the AmeriSpeak panel were reported elsewhere.[1] The sample for this study was selected from the panel using sampling strata based on age, race/ethnicity, gender, and education (with a total of 48 sampling strata). The sample size per stratum was determined based on the population distribution for each stratum. The sample selection considered the expected differential survey complete rates by demographic groups. The surveys were conducted both online and via telephone, using a 15-minute questionnaire. Data were collected from January 10-28, 2022, resulting in 2,598 completed responses (completion rate 30.1%, with 2,508 collected by web mode, and 90 by phone mode). Participants were offered the cash equivalent of $3 for completing the survey.

Post-hoc Weighting

The survey weights were adjusted for non-response, and then further adjusted to the external population totals via a raking ratio method. The post-stratification weighting variables that were used included age, gender, census division, race/ethnicity, education, age*gender, age*race/ethnicity, and race/ethnicity*gender. The external population totals for the weighting variables were obtained from the Current Population Survey (conducted by the United States Census Bureau). Raking and re-raking was done during the weighting process such that the weighted demographic distributions of the survey respondents resemble the demographic distributions of the target population (i.e., U.S. adults aged 18 years and older within all 50 states and the District of Columbia).

Measurement

Demographic factors. Age was categorized into the following groups based on birth cohorts: Gen Z (18-25 years), Millennial (26-41 years), Gen X (42-57 years), Boomer (58-76 years), and Silent Generation (77+ years). Race/ethnicity included the following groups: non-Hispanic Asian, non-Hispanic Black, non-Hispanic White, non-Hispanic two or more races, non-Hispanic Other, and Hispanic. Gender included female and male.

© 2022 Chen Y et al. *JAMA Psychiatry.*

Wellbeing. Wellbeing (or flourishing) is sometimes understood as “a state in which all aspects of a person’s life are good”.[2] With this conceptualization of wellbeing, VanderWeele proposed a theoretical framework of human flourishing considering multiple domains that are often conceived as essential to human life.[2] These include happiness and life satisfaction, physical and mental health, meaning and purpose, character and virtue, and close social relationships. These domains each satisfy two criteria including: 1) it is nearly universally desired, and 2) it is generally considered as an end in itself. Although financial and material stability are generally not considered as ends for pursuit, sufficient resource stability can be an important precondition for sustaining well-being in other domains. Therefore, VanderWeele also proposed an additional “enabling” domain of financial and material stability. Based on this theoretical framework of human flourishing, VanderWeele proposed a 12-item secure flourishing index[2] that measures wellbeing in each of the 6 domains and the overall wellbeing across these domains. All items (see below for item wording) were self-reported on a scale 0-10. Financial and material stability item wording was effectively reverse coded, such that a higher score indicates greater wellbeing. Domain scores were derived by averaging the two indicators under each domain; the overall wellbeing score averaged indicator responses across domains. This proposed index has been validated previously, and showed evidence of good psychometric properties (including evidence of goodness of fit, satisfactory reliability, good test-retest correlation, adequate construct validity, and acceptable measurement invariance).[3]

The secure flourishing index

1. How satisfied are you with your life as a whole these days?
2. How happy or unhappy do you usually feel?
3. How would you rate your physical health?
4. How would you rate your overall mental health?
5. To what extent do you feel the things you do in your life are worthwhile?
6. I understand my purpose in life.
7. I always act to promote good in all circumstances, even in difficult and challenging situations
8. I am always able to give up some happiness now for greater happiness later
9. I am content with my friendships and relationships.
10. My relationships are as satisfying as I would want them to be.
11. How frequently do you worry about being able to meet normal monthly living expenses?
12. How frequently do you worry about safety, food, or housing?

† Each question or statement is evaluated 0-10. Anchors are:

Q1 (0=Not Satisfied at All, 10=Completely Satisfied); Q2 (0=Extreme Unhappy, 10=Extremely Happy); Q3 and Q4 (0=Poor, 10=Excellent); Q5 (0=Not at All Worthwhile, 10=Completely Worthwhile); Q6, Q9, and Q10 (0=Strongly Disagree, 10=Strongly Agree); Q7 and Q8 (0=Not True of Me, 10=Completely True of Me); Q11 and Q12 (0=Worry All of the Time, 10=Do Not Ever

© 2022 Chen Y et al. *JAMA Psychiatry.*

Worry). Q1 and Q2 constitute the Happiness & Life Satisfaction domain; Q3 and Q4 Mental & Physical Health; Q5 and Q6 Meaning & Purpose; Q7 and Q8 Character & Virtue; Q9 and Q10 Close Social Relationships; and Q11 and Q12 Financial & Material Stability. The Secure Flourishing Index is an average of the responses from Q1 through Q12 and include the Financial and Material Stability domain. Reliability for the secure flourishing index in cross-cultural samples is alpha=0.86.

Analyses Assumptions

While not all of the scores of the 12 individual flourishing items follow normal distribution, with a large sample size as in this case, the mean values themselves will be approximately normal according to the central limit theorem.

Missing Data

There was no missing data on demographic variables. Approximately 95.50% of the participants (2,481 out of 2,598 participants) had complete data on all flourishing items, with missing data on the 12 individual flourishing items ranging from 0.27% to 0.77% (i.e., the number of participants with missing data on the individual flourishing items ranged from 7 to 20 out of 2,598 participants).

Multiple imputation by chained equations (with 5 imputed datasets)[4] was used to impute missing data on the flourishing items. The following variables were included in the imputation model: the 12 individual flourishing items, age, gender, race/ethnicity, marital status, U.S. census region, educational level, household income, and religious service attendance. Complete-case analyses yielded similar results as the primary analyses with imputation for missing data.

References for the Supplement

1. NORC at the University of Chicago. Technical overview of the AmeriSpeak panel NORC's probability-based household panel. Updated February 8, 2022. Accessed May 1, 2022. https://amerispeak.norc.org/content/dam/amerispeak/research/pdf/AmeriSpeak%20Technical%20Overview%202019%2002%2018.pdf
2. VanderWeele TJ. On the promotion of human flourishing. *Proc Natl Acad Sci U S A*. Aug 1 2017;114(31):8148-8156. doi:10.1073/pnas.1702996114
3. Weziak-Bialowolska D, Bialowolski P, Lee MT, Chen Y, VanderWeele TJ, McNeely E. Psychometric Properties of Flourishing Scales From a Comprehensive Well-Being Assessment. *Front Psychol*. 2021;12:652209. doi:10.3389/fpsyg.2021.652209
4. Sterne JA, White IR, Carlin JB, et al. Multiple imputation for missing data in epidemiological and clinical research: potential and pitfalls. *BMJ*. Jun 29 2009;338:b2393. doi:10.1136/bmj.b2393

© 2022 Chen Y et al. *JAMA Psychiatry.*
